# Medical Students and Suicide Prevention: Training, Education, and Personal Risks

**DOI:** 10.3389/fpsyg.2018.00452

**Published:** 2018-04-09

**Authors:** Carla Gramaglia, Patrizia Zeppegno

**Affiliations:** ^1^Psychiatry Institute, Dipartimento di Medicina Traslazionale, Università degli Studi del Piemonte Orientale, Novara, Italy; ^2^Struttura Complessa di Psichiatria, Azienda Ospedaliero Universitaria Maggiore della Carità di Novara, Novara, Italy

**Keywords:** medical students, suicide, prevention, risk, education, training

Exposure to suicidal patients is widespread among psychiatrist and psychiatric nurses, but also among general practitioners, emergency clinicians, and nurses (Palmieri et al., 2008). Patients who attempt suicide are more likely to have contact with their primary care provider than a mental health provider in the month before attempting suicide (Lake, 2008). Hence, it will often be up to medical personnel, beyond psychiatrists, to encounter suicide attempters. Regrettably, depression and the related risk for suicide are still largely underrecognized in primary care settings and emergency rooms (Lake, 2008). To improve diagnostic skills and competency in suicide ideation (SI) assessment, specific training is needed because it could greatly contribute to suicide prevention (Palmieri et al., 2008). Educational strategies to increase SI assessment performance should be available to all professions involved in general and psychosocial patient care (Mospan et al., 2017), starting from medical and residency schools.

These educational strategies usually address theoretical and technical skills, but a complementary facet deserving attention is attitude toward suicide. Therefore, besides increasing theoretical competency in depression and suicide risk assessment, there should be also a specific focus on medical students' and clinicians' attitudes toward these constructs. Attitudes may have a dual root: one related to clinical experience, and the other to personal experience.

Regarding the first, the majority of health professionals have unfavorable attitudes toward patients' self-harm, which might compromise their therapeutic endeavors and outcomes. Several studies have investigated this topic in medical students, using different tools (Oncü et al., 2008; Palmieri et al., 2008; Yousuf et al., 2013; Chan et al., 2014; Hashimoto et al., 2014; Nebhinani et al., 2016), and have suggested an association between medical students' attitudes toward suicide and appropriate therapeutic responses to suicidal individuals (Hashimoto et al., 2014). Students' knowledge and attitudes toward suicide have been described as changing during the years of training. Sato and coworkers assessed medical students in their first, third, and fifth year, with multiple-choice questions. In the knowledge part, only about half of the items were answered correctly; a significant difference was observed in students' attitudes, with sympathetic comments increasing, and critical comments decreasing, along with student years (Sato et al., 2006).

As far as personal experience is concerned, it has been suggested that attitude toward patients' depression and suicidal behaviors may also be related to depression and suicide risk among medical students and practitioners. A growing body of literature documents high rates of burnout, depression, and suicidal ideation among physicians and medical students (Torre et al., 1999, 2000, 2001; Rotenstein et al., 2016). Many medical students experience distress during medical school: the training process and environment themselves could contribute to the deterioration of students' mental health (Brazeau et al., 2014), and a relationship between burnout and suicidal thoughts has been suggested (van der Heijden et al., 2008).

A recent review and meta-analysis reported that the summary estimate of the prevalence of depression or depressive symptoms among medical students was 27.2% and that of suicidal ideation was 11.1% (Rotenstein et al., 2016). Nonetheless, only 15.7% of depressed medical students sought psychiatric treatment (Rotenstein et al., 2016). Puthran et al. (2016) reported similar rates for depression (28%) and proportion of treatment-seeking depressed students (12.9%), but a lower mean frequency of suicide ideation (5.8%). Another study identified older students and those of lower socio-economic status as the main target of suicide intervention programmes and depression counseling (Fan et al., 2012).

What described above, as regards both clinical and personal experience, unveils another relevant problem, i.e., stigma. While psychiatrists may be familiar with this construct in their clinical experience, they may be less aware of its impact on treatment-seeking behaviors in the category of medical students and workers. Actually, strong concerns about stigma, confidentiality, adverse effects on residency application, and potential career impact have been reported. These may lead to barriers to mental health treatment, and explain the low rate of students seeking psychiatric treatment despite screening positive for depression reported above (Puthran et al., 2016; Rotenstein et al., 2016). Another contribution to barriers to seeking appropriate care include inadequate education about causes, effects, and treatment, unwillingness to take the needed time, and limited financial resources to pay for care (Reynolds and Clayton, 2009; Downs et al., 2014).

It is worrisome that many students share beliefs like the following: that revealing depression could negatively affect their professional advancement (Wimsatt et al., 2015); that if they were depressed their opinions would be respected less; that they would be considered unable to handle their responsibilities, and “less intelligent” (Schwenk et al., 2010). These beliefs have been reported to differ by sex; for instance, men have been found to agree more commonly than woman that depressed students could endanger patients (Schwenk et al., 2010). A US study identified three constructs underlying stigma: personal weakness, public devaluation, and social/professional discrimination. Students associating personal weakness with depression perceived medication as less efficacious and the academic environment as more competitive (Wimsatt et al., 2015).

The stigma problem deserves to be addressed, since as medical students and physicians confront depression and suicidality in their peers, they may become more likely to recognize and treat these conditions in patients, including colleagues (Center et al., 2003). Albeit it may not reflect objective measures of competency, a survey of Australian medical students found an association between greater personal experience of suicide and previous contact with patients with psychiatric problems and both increased perceived comfort and increased confidence in providing care for individuals with suicidal thoughts or behaviors (Patel et al., 2016). On the other hand, Chan et al. (2014) found that less exposure to suicidal people through clinical experience was associated with greater stigma and increased intentions of informal help seeking on behalf of students, while those who normalized suicide had significantly lower intentions of seeking help for thoughts of suicide. According to these two studies, greater personal experience of suicide and more exposure to patients with psychiatric problems and suicidal behaviors may have a two-fold impact: on the one hand it could increase confidence in providing care to patients (Patel et al., 2016) and decrease stigma (Chan et al., 2014), while on the other it could lead to an enantiodromic attitude (“normalization” of suicide) and to a decreased intention of help seeking for personal problems including suicidal thoughts (Chan et al., 2014).

Briefly, further studies assessing suicide prevention curricula and interventions aimed at raising mental health literacy levels are warranted (Hawgood et al., 2008; Yousuf et al., 2013; Cheng et al., 2014) to increase awareness of depression and to destigmatize help-seeking in order to prevent suicide (Reynolds and Clayton, 2009; Moutier et al., 2012), both in the overall population of patients, and specifically in the medical students' and clinicians' category.

A question emerges here about how to properly approach these complex issues. According to what has been described above, personal experience alone is not enough, as well as the clinical one. Training and theoretical education may not be enough as well.

Some suggestions can be found in the archetype of the wounded healer (Guggenbühl-Craig, 1999), which teaches that within the physician there is the healer as well as the patient. Mythological references to this phenomenon include Aesculapius's teacher, Chiron, who suffered from incurable wounds (Greece), and Kali, goddess of pox but curer as well (India). Nonetheless, while “Knowing your own darkness is the best method for dealing with the darknesses of other people” (Jung, 1973), it is not enough: “who bears a wound could better empathize, who has experienced a similar sufference could better understand, but the wounded healer embodies a different type of consciousness” (Torre, 2010).

This “different type of consciousness” is not easy to achieve. In one of his Letters, Carl Gustav Jung wrote: “You can learn a great deal of psychology through studying books, but you will find that this psychology is not very helpful in practical life,” because you need “not only a piece of knowledge, but a certain wisdom of life.” Wisdom of life “does not consist of words only but chiefly of experience”: “If such a thing can be taught at all, it must be in the way of a personal experience of the human soul. Such an experience is possible only when the teaching has a personal character, namely when you are personally taught and not generally.”

It clearly emerges the need of a thorough approach encompassing both knowledge and attitude, with the two-fold purpose of increasing competencies and allowing a reflection on personal attitudes and emotions. In the field of education, it is widely acknowledged that communication and sharing of feeling-related subjects are complex to characterize. They belong to the field of personal knowledge (attitude), rather than to that of theoretical and practical knowledge (know that and know-how). While advances in the teaching of technical knowledge are going at the same pace of technical innovation itself, the same cannot be stated for the field of personal knowledge (Torre, 2007; Torre et al., 2017). Wilfred Bion realized that, contrary to technical issues, which are easy to share and teach, only limited methods exist to communicate emotional experiences. In this field, a mimetic approach is not only worthless, but also dangerous because it might resemble a spurious appearance of growth (Bion, 1948).

Our Psychiatry School has a longstanding experience in the education and training of medical students and clinicians, and also in the counseling of students (a free Counseling service for students exists in Our University since 1988, when it was founded by Eugenio Torre). The theoretical root of our approach is the method Eugenio Torre developed trying to find a possible answer to the unresolved question raised by Bion. This innovative approach uses dynamic images (full length movies or scenes) from cinematic fiction as educational incitements, and combines theoretical and technical issues together with the experience of working in a group, also with the use of role play (Torre, 2007; Gramaglia et al., 2013; Zeppegno et al., 2015, 2017; Torre et al., 2017).

Approaches as arts-based therapy (including cinema among the arts) and role play, can be used as a valuable means of expression and exploration of feelings, to enhance personal growth and change, acting more on an emotional level than an intellectual one. Moreover, such approaches have the potential to help the individual grow in self-awareness while feeling relatively “safe” and protected by the safeguard distance offered by a movie or movie scene or by the role play (Izod, 2000). This feeling of being relatively “safe” may be particularly important when discussing about suicide.

Some Authors have used simulation and standardized patients or actors to approach the complex topic of suicidal behaviors (Fiedorowicz et al., 2013; Foster et al., 2015), nonetheless we believe that interactive learning techniques with the direct involvement of students may be particularly relevant to educate about complex topics which elicit strong personal and emotional responses, as is the case for suicidal behaviors. Actually, our experience is that role play with students and trainees in the role of both patients and clinicians has a great potential to enhance reflection about their emotions and improve their communication skills.

As we have tried to outline in this paper, there is still work to do in the education about suicide offered to medical students, clinicians and psychiatrists. While the gaps in theoretical and technical skills may be easier to fill, more attention should be paid to students' and clinicians' attitudes toward suicide, to the bi-directional relation between attitudes and experience (both clinical and personal), and to stigma. New educational approaches, complementary to the “traditional” ones, should be sought for this purpose, and we have briefly outlined our experience with the method proposed by Eugenio Torre. An approach including the arts (mythology, literature, cinema…), may represent for students and clinicians a privileged pathway to allow a reflection on the possibility of illness (including psychiatric illness), and death (including self-inflicted death), which are inherent in their human being. This may help develop that “different type of consciousness” described above: “Otherwise everything remains a clever intellectual trick, consisting of empty words and leading to empty talk (Torre, 2010).”

## Author contributions

CG: performed the literature search; CG and PZ wrote the manuscript.

### Conflict of interest statement

The authors declare that the research was conducted in the absence of any commercial or financial relationships that could be construed as a potential conflict of interest.
